# Self-Produced Time Intervals Are Perceived as More Variable and/or Shorter Depending on Temporal Context in Subsecond and Suprasecond Ranges

**DOI:** 10.3389/fnint.2016.00019

**Published:** 2016-06-01

**Authors:** Keita Mitani, Makio Kashino

**Affiliations:** ^1^Department of Information Processing, Tokyo Institute of TechnologyYokohama, Japan; ^2^Human Information Science Laboratory, NTT Communication Science Laboratories, Nippon Telegraph and Telephone CorporationAtsugi, Japan

**Keywords:** time perception, motor timing, subsecond timing, suprasecond timing, body movements, weber fraction, contextual effects, auditory

## Abstract

The processing of time intervals is fundamental for sensorimotor and cognitive functions. Perceptual and motor timing are often performed concurrently (e.g., playing a musical instrument). Although previous studies have shown the influence of body movements on time perception, how we perceive self-produced time intervals has remained unclear. Furthermore, it has been suggested that the timing mechanisms are distinct for the sub- and suprasecond ranges. Here, we compared perceptual performances for self-produced and passively presented time intervals in random contexts (i.e., multiple target intervals presented in a session) across the sub- and suprasecond ranges (Experiment 1) and within the sub- (Experiment 2) and suprasecond (Experiment 3) ranges, and in a constant context (i.e., a single target interval presented in a session) in the sub- and suprasecond ranges (Experiment 4). We show that self-produced time intervals were perceived as shorter and more variable across the sub- and suprasecond ranges and within the suprasecond range but not within the subsecond range in a random context. In a constant context, the self-produced time intervals were perceived as more variable in the suprasecond range but not in the subsecond range. The impairing effects indicate that motor timing interferes with perceptual timing. The dependence of impairment on temporal contexts suggests multiple timing mechanisms for the subsecond and suprasecond ranges. In addition, violation of the scalar property (i.e., a constant variability to target interval ratio) was observed between the sub- and suprasecond ranges. The violation was clearer for motor timing than for perceptual timing. This suggests that the multiple timing mechanisms for the sub- and suprasecond ranges overlap more for perception than for motor. Moreover, the central tendency effect (i.e., where shorter base intervals are overestimated and longer base intervals are underestimated) disappeared with motor timing within the subsecond range, suggesting multiple subsecond timing system for perception and motor.

## Introduction

Time interval processing is essential for sensorimotor and cognitive function (Mauk and Buonomano, 2004; Buhusi and Meck, 2005). The perceptual and motor aspects of temporal processing are often entangled. For example, when playing a musical instrument, a player must perceive self-produced time intervals and use the perception to adjust the timings of musical notes produced by their own actions. The perception of time intervals presented actively (i.e., self-produced) must be accompanied by body movements and timing processing for motor control, which differ from that of time intervals presented passively. If body movements and timing processing for motor control influence time perception, we must perceive self-produced time intervals differently from passively presented time intervals.

It is known that body movements affect time perception, and the effect depends on the situation. One effect is the improvement of time perception. In the visual and tactile modalities, body movements can also reduce temporal illusions induced by stimulus movements (Tomassini et al., 2012; Carlini and French, 2014). In the auditory but not the visual modality, the initiation of time intervals by voluntary button pressing can improve temporal sensitivity (Iordanescu et al., 2013). The synchronization of body movements to musical beats can improve temporal sensitivity (Manning and Schutz, 2013). These two studies suggest that auditory-motor coupling benefits time perception when target intervals follow action. If auditory-motor coupling benefits time perception even when the target intervals are determined by the listener’s own action, it is expected that self-produced time intervals will be perceived more accurately than passively presented time intervals in the auditory modality.

In contrast, body movements can distort subjective time. For instance, an intentional action can shorten the subjective time interval between the action and its sensory consequence, which is referred to as intentional binding (Haggard et al., 2002; Wenke and Haggard, 2009). On the other hand, the subjective time interval of visual stimuli during rapid hand movements can be compressed (Yokosaka et al., 2015). Although, previous studies have shown the influence of body movements on time perception, the case where the target intervals are marked by the observer’s own action has not been investigated. Hagura et al. (2012) have shown that the time interval preceding action is lengthened subjectively. Thus, if only body movements are considered to be a factor influencing time perception, self-produced time intervals would be lengthened subjectively.

On the other hand, whether and how timing processing for motor control affects time perception have been less investigated, although whether the mechanisms of motor and perceptual timing are common or distinct has been debated (Keele et al., 1985; Ivry and Hazeltine, 1995; Ivry, 1996; Meegan et al., 2000; Schubotz et al., 2000; Macar et al., 2002; Repp, 2002; Lewis and Miall, 2003b; Mauk and Buonomano, 2004; Buonomano, 2005; Bueti et al., 2008; Bueti and Walsh, 2010; Merchant et al., 2008; Wiener et al., 2010; Bangert et al., 2011). Although previous studies have shown a visually guided motor task requiring timing processing for motor control to impair time perception (Brown, 1985, 1997; Hass et al., 2012), no one has asked whether temporal reproduction affects time perception. If the effects of a visually guided motor task and temporal reproduction are the same, it is expected that self-produced time interval will be perceived as more variable.

It is possible that whether target intervals are in the sub- or suprasecond range affects the influence of a self-producing time interval, because it has been proposed that the mechanisms of sub- and suprasecond timing are distinct (Lewis and Miall, 2003b; Mauk and Buonomano, 2004; Buhusi and Meck, 2005; Ivry and Schlerf, 2008; Grondin, 2010). It has been shown that pharmacological manipulations affect time perception differently in the sub- or suprasecond range (Meck, 1996; Rammsayer, 1999; Coull et al., 2011). Additionally, neuroimaging studies have shown that neural sites activated by timing tasks depend on whether the target time interval is in the sub- or suprasecond range (Lewis and Miall, 2003a,b; Wiener et al., 2010). Recent meta-analyses have indicated that temporal processing in the subsecond range tends to require subcortical activation including the cerebellum and basal ganglia, whereas that in the suprasecond range tends to require cortical activation including the supplementary motor area and prefrontal cortex (Wiener et al., 2010). A voxel-based morphometry study has also shown that the performance of sub- and suprasecond time perception tasks is correlated with gray matter volume in the cerebellum and in the inferior parietal cortex, respectively (Hayashi et al., 2014). These neural substrates associated with time perception suggest that interval timing in the subsecond range is automatic and that in the suprasecond range is cognitively mediated. Indeed, previous studies using a dual-task paradigm have shown that the interference effect of a concurrent non-temporal cognitive task on a timing task is greater when the target interval is in the suprasecond range than in the subsecond range (Rammsayer and Lima, 1991; Miyake et al., 2004; Rammsayer and Ulrich, 2011; Maes et al., 2015; but see Rammsayer and Ulrich, 2005). Therefore, timing mechanisms are expected to require attentional resources in the suprasecond range but not in the subsecond range. We hypothesized that timing processing for motor control interferes with perceptual timing in the suprasecond range but not in the subsecond range.

**Figure 1:**
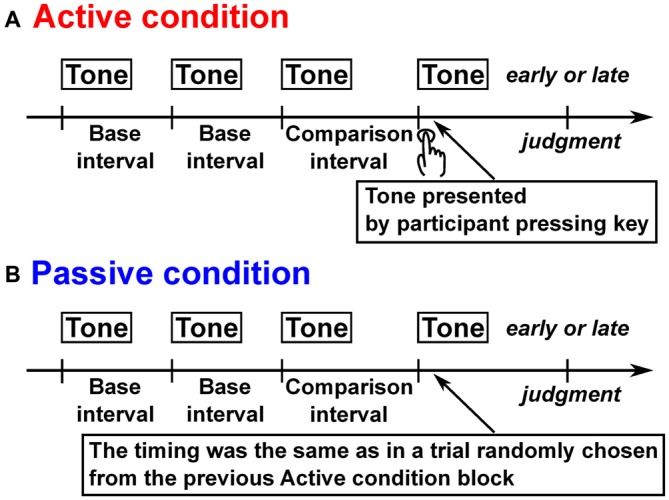
**Schematic illustration of trial structure in all experiments. (A)** In the active condition, after three isochronous tones were presented, participants presented the last tone by pressing a key to make the entire tone sequence isochronous, and judged whether the last tone was early or late from the isochronous timing. **(B)** In the passive condition after four tones were presented whose first three were isochronous with no participants’ movement, the participants judged whether the last tone was early or late from the isochronous timing.

This study examined whether and how a self-producing time interval affects time perception in the sub- and suprasecond ranges. As mentioned above, a self-produced time interval is related to body movements and timing processing for motor control, which can influence time perception. If the effect of body movements is dominant, self-produced time intervals are perceived as more accurate and longer. In contrast, if the effect of timing processing for motor control is dominant, self-produced time intervals are perceived as more variable in the suprasecond range but not in the subsecond range. To investigate this, we measured the criterion and accuracy of time interval judgments when the target interval to be determined was terminated by participant’s own action (active condition), and when the target interval was presented passively (passive condition; see Figure 1). The experiment was conducted under various random contexts (i.e., multiple target intervals were presented in a session) where the target intervals is across the sub- and suprasecond ranges (Experiment 1), within the subsecond range (Experiment 2), and within the suprasecond range (Experiment 3). To assess contextual effects, we performed separate measurements in the sub- and suprasecond ranges in a constant context (i.e., a single target interval was presented in a session; Experiment 4).

## Experiment 1

### Materials and Methods

#### Participants

Fourteen individuals participated in the experiment. One of the participants was the first author. All the participants had normal hearing and were right-handed. With the exception of the author, the participants gave written informed consent and were paid for their participation. All experiments were conducted in accordance with the Declaration of Helsinki and were approved by the Ethics and Safety Committees of NTT Communication Science Laboratories (Atsugi, Japan). Data for 3 of the 14 participants were excluded (see “Analysis” Section). The data obtained from the remaining 11 participants (10 female, average age 34.5 years, *SD* = 5.6 years) were analyzed.

#### Apparatus

The experiment was conducted in a sound-insulated booth. Stimulus presentation and data acquisition were performed by a computer [Apple; Mac Book Air (11 inch, Mid 2013)] using MATLAB 8.1 (The MathWorks) and Psychophysics Toolbox Version 3 (Kleiner et al., 2007). The stimuli were presented through a digital audio interface (Roland; UA-25EX) and headphones (Sennheiser; HDA200). The sampling frequency was 44.1 kHz.

#### Stimuli and Procedure

The experiment was conducted under an active condition where the time interval was reproduced by the participant’s own action after listening to a tone sequence, and also under a passive condition, in which a tone sequence was presented passively (see Figure 1). Each trial started with the presentation of a pure tone (duration: 50 ms, rise/fall: 10 ms, frequency: 2 kHz, sound pressure level: about 80 dB) to inform the participants that a trial was beginning. In the active condition, after a 6 s delay, three successive isochronous pure tones (duration: 50 ms, rise/fall: 10 ms, frequency: 1 kHz, sound pressure level: about 80 dB) were presented whose interonset intervals were chosen randomly from 0.5, 1.2, 2.2, 3.2, and 4.2 s. After the presentation, the participants generated the same tone as the previous tones by pressing a key in an effort to make the entire tone sequence as isochronous as possible. The participants judged whether the last self-produced tone was early or late relative to the isochronous timing of the other tones in the sequence. In the passive condition, they listened to four successive tones with the same intervals as in a trial chosen randomly from the previous active condition block, and judged whether the last tone was early or late as in the active condition. The next trial started after a 1 s delay.

The participants were asked to close their eyes, not to use the strategy of subdividing intervals, and not to move their bodies rhythmically during the listening tone sequence. It is known that the strategy of subdividing intervals improves the temporal discrimination in the suprasecond range (Grondin et al., 1999). They were also asked to use their right index fingers in contact with a key to reproduce the intervals.

Each block consisted of 12 trials for each base interval. A passive condition block was always after an active condition block. Each block was separated by a rest. The experiment was performed over 2 days. There were a total of 14 sessions (7 sessions per day). The first session on each day was excluded from the analysis. Thus, 12 × 12 = 144 data were obtained for each condition/base interval/participant.

#### Analysis

**Figure 2:**
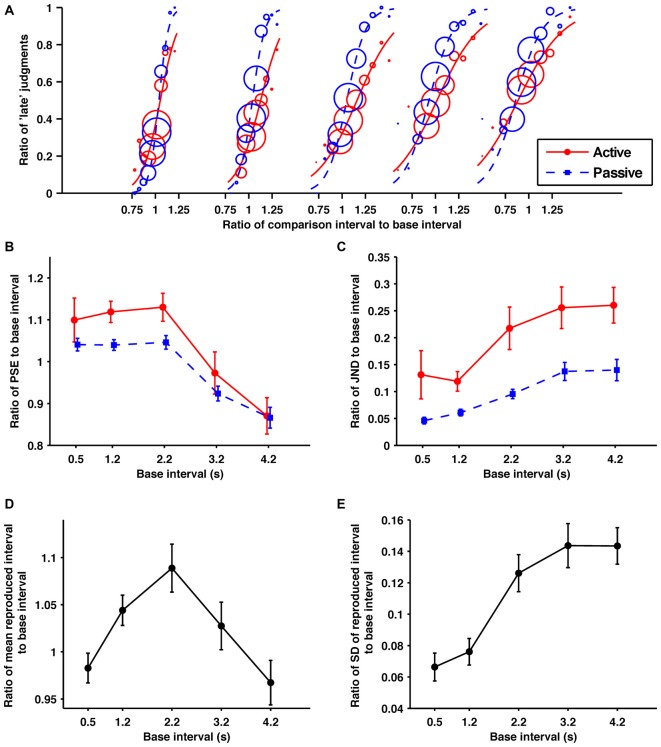
**Across the sub- and suprasecond ranges the self-produced time intervals were perceived as shorter and more variable. (A)** Probability of judgments calculated from the data collected from all the participants, and the fitted psychometric curves in each condition/base interval. The red solid and blue dashed lines indicate in the active and passive conditions, respectively. In order from the left, these fitted psychometric curves are for 0.5, 1.2, 2.2, 3.2, and 4.2 s base intervals. The size of circle reflects the number of trials at each comparison interval. **(B)** Averaged data for the ratios of points of subjective equality (PSEs), **(C)** just noticeable differences (JNDs)** (D)** means of reproduction** (E)** standard deviations (SDs) of reproduction to base interval as a function of base interval. The main effects of condition for PSEs and JNDs were significant. All the main effects of base interval were significant. Error bars represent the standard error of the mean.

Before the data analysis, data whose last interval was less than 2 inter-quartile ranges (IQRs) from the first quartile or more than 2 IQRs from the third quartile were excluded as errors. A logistic regression by the maximal likelihood method was used to estimate the percentage of “late” judgment responses to the last interval for each condition/base interval/participant. The fitted psychometric curves for the response data aggregated from all the participants are shown in Figure 2A. The fitting was conducted before the subject responses were sorted into bins. The point of subjective equality (PSE), which was defined as the last interval corresponding to a 50% “late” response rate, and the just noticeable difference (JND), which was defined as half of the difference between the last intervals corresponding to 25% and 75% “late” response rates, were calculated from each regression as indexes of the criterion and precision of judgments, respectively. On the other hand, the mean and standard deviation (SD) of the last intervals were calculated as indexes of the reproduction performance. The ratios of these indexes to their base intervals were computed to make it possible to compare them for different base interval conditions. Three participants were excluded, because the estimated probability curves of their judgments were reversed in the active condition in one or more base intervals.

For a statistical analysis, two-way (condition × base interval) and one-way (base interval) repeated-measures analysis of variances (ANOVAs) were performed. For all *post hoc* comparisons, paired *t*-tests with the Holm-Bonferroni correction were employed. The degrees of freedom were adjusted with the Greenhouse-Geisser epsilon whenever appropriate.

### Results and Discussion

#### Discrimination Performance

The obtained ratios of the PSEs to their base intervals are shown in Figure 2B. A 2 (condition; active and passive) × 5 (base interval; 0.5, 1.2, 2.2, 3.2, 4.2 s) repeated-measures ANOVA of these data revealed a significant main effect of condition (*F*_(1,10)_ = 8.19, *p* = 0.017, $\eta_{p}^{2}$ = 0.450) and base interval (*F*_(1.5,14.5)_ = 12.04, *p* = 0.002, $\eta_{p}^{2}$ = 0.546). The interaction was not significant (*p* = 0.45). The significant main effect of condition suggests that temporal reproduction shortens comparison intervals subjectively. *Post hoc* paired *t*-tests with the Holm-Bonferroni correction revealed that the ratios of PSEs to their base intervals for the 0.5, 1.2, 2.2, and 3.2 s base intervals were larger than for the 4.2 s base interval, and those for the 1.2 and 2.2 s base intervals were larger than for the 3.2 s base interval (*p* < 0.05 for all comparisons). No other differences reached the 5% level of statistical significance. These results indicate that generally the shorter base intervals were overestimated and the longer base intervals were underestimated. This tendency was consistent with previous studies, and has been referred to as the central tendency effect (for a review see, Shi et al., 2013).

The obtained ratios of the JNDs to their base intervals are shown in Figure 2C. A 2 (condition) × 5 (base interval) repeated-measures ANOVA of these data revealed a significant main effect of condition (*F*_1,10_ = 23.45, *p* < 0.001, $\eta_{p}^{2}$ = 0.701) and base interval (*F*_(2.5,25.0)_ = 9.59, *p* < 0.001, $\eta_{p}^{2}$ = 0.490). The interaction was not significant (*p* = 0.41). The significant main effect of condition suggests that the temporal reproduction worsens temporal sensitivity. *Post hoc* paired *t*-tests with the Holm-Bonferroni correction revealed that the temporal sensitivity for the 0.5 s base interval was better than for 3.2 and 4.2 s base intervals, and that for the 1.2 s base interval was better than for 2.2, 3.2, and 4.2 s base intervals (*p* < 0.05 for all comparisons). No other differences reached the 5% level of statistical significance. These results indicate that the temporal sensitivity gradually worsened as the base interval became longer.

#### Reproduction Performance

The obtained ratios of the means to their base intervals are shown in Figure 2D. A one-way repeated-measures ANOVA of these data revealed a significant main effect (*F*_(2.12,21.2)_ = 13.49, *p* < 0.001, $\eta_{p}^{2}$ = 0.574). *Post hoc* paired *t*-tests with the Holm-Bonferroni correction revealed that the ratio of the mean of reproduction to its base interval for the 0.5 s base interval was smaller than for the 1.2 and 2.2 s base intervals, that for the 2.2 s was larger than for the 3.2 s base interval, and those for the 1.2, 2.2 and 3.2 s base intervals were larger than for the 4.2 s base interval (*p* < 0.01 for all comparisons). No other differences reached the 5% level of statistical significance. From these results, the central tendency effect on motor timing seems to exist in the suprasecond range but not in the subsecond range. This is consistent with previous research, which has shown the central tendency effect on auditory motor timing is negligible in the subsecond range (Repp, 2002; Cicchini et al., 2012).

The obtained ratios of the SDs to their base intervals are shown in Figure 2E. A one-way repeated-measures ANOVA of these data revealed a significant main effect (*F*_(1.7,16.6)_ = 27.43, *p* < 0.0001, $\eta_{p}^{2}$ = 0.733). *Post hoc* paired *t*-tests with the Holm-Bonferroni correction revealed that the ratios of SDs to their base intervals for the 0.5 and 1.2 s base intervals were smaller than for the 2.2, 3.2 and 4.2 s base intervals (*p* < 0.01 for all comparisons). No other differences reached the 5% level of statistical significance. These results suggest that the variability of motor timing increased suddenly between 1.2 and 2.2 s. This violation of the scalar property is consistent with perceptual timing. However, its sharpness is quite different for motor and perceptual timing.

## Experiment 2

In experiment 1, we showed that the self-produced time intervals were perceived as more variable and shorter than passively presented time intervals across the sub- and suprasecond ranges. However, it is possible that the temporal contexts in which the target intervals are presented across the sub- and suprasecond ranges affect the self-produced time intervals. Therefore, we conducted experiments that were identical to experiment 1 except as regards the base intervals. In experiment 2, the base intervals were in the subsecond range.

### Materials and Methods

The experimental settings were the same as for experiment 1. Fifteen individuals participated in the experiment. The base intervals used in this experiment were 0.3, 0.4, 0.5, and 0.6 s. Data obtained for 4 of the 15 participants were excluded. The reasons for exclusion were that the estimated probability curves of the judgments of two of the four participants were reversed in the active condition in one or more base intervals, and the ratio of PSE to its base interval of another two was extremely high (>2) in one or more conditions. The data obtained for the remaining 11 participants (7 female, average age 36.3 years, *SD* = 5.7 years) were analyzed.

### Results and Discussion

#### Discrimination Performance

**Figure 3:**
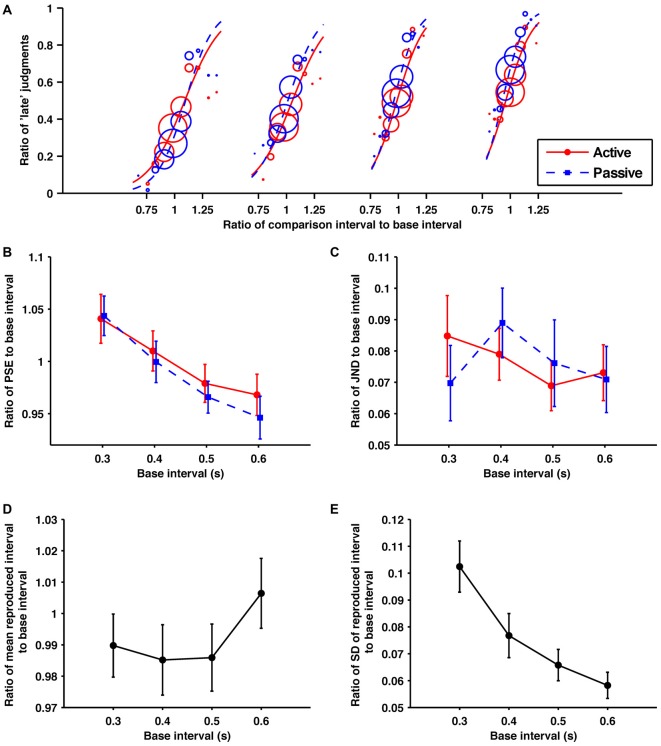
**Within the subsecond range, the self-produced time intervals were perceived as similar to passively presented time intervals. (A)** Probability of judgments calculated using the data collected from all participants, and the fitted psychometric curves in each condition/base interval. In order from the left, these fitted psychometric curves are for base intervals of 0.3, 0.4, 0.5 and 0.6 s. **(B)** Averaged data for the ratios of PSEs, **(C)** JNDs, **(D)** means of reproduction **(E)** SDs of reproduction to base interval as a function of base interval. The main effects of condition for PSEs and JNDs were not significant. The main effects of base interval for PSEs, means and SDs of reproduction were significant. Error bars represent the standard error of the mean.

The fitted psychometric curves for the response data aggregated from all the participants are shown in Figure 3A. The obtained ratios of the PSEs to their base intervals are shown in Figure 3B. A 2 (condition; active and passive) × 4 (base interval; 0.3, 0.4, 0.5, 0.6 s) repeated-measures ANOVA of these data revealed a significant main effect of base interval (*F*_(1.27,12.7)_ = 13.10, *p* = 0.002, $\eta_{p}^{2}$ = 0.567). The main effect of condition and the interaction was not significant (*p* = 0.16, *p* = 0.58, respectively). In contrast to experiment 1, the PSEs showed no effect of temporal reproduction. *Post hoc* paired *t*-tests with the Holm-Bonferroni correction revealed that the ratio of PSE to its base interval for the 0.3 s base interval was larger than for all longer base intervals. That for the 0.4 s base interval was larger than for all longer base intervals, and that for the 0.5 s base interval was larger than for the 0.6 s base interval (*p* < 0.05 for all comparisons). These results clearly illustrate the central tendency effect on perceptual timing in the subsecond range.

The obtained ratios of the JNDs to their base intervals are shown in Figure 3C. A 2 (condition) × 5 (base interval) repeated-measures ANOVA of these data was performed. The main effects of condition, base interval, and the interaction were not significant (*p* = 1.00, *p* = 0.16, *p* = 0.19, respectively). In contrast to experiment 1, the temporal reproduction was not found to have any effect on the precision as well as the PSEs in this experiment.

#### Reproduction Performance

The obtained ratios of the means to their base intervals are shown in Figure 3D. A one-way repeated-measures ANOVA of these data revealed a significant base interval effect (*F*_(2.17,21.7)_ = 5.26, *p* = 0.012, $\eta_{p}^{2}$ = 0.345). *Post hoc* paired *t*-tests with the Holm-Bonferroni correction revealed that the ratio of the mean to its base interval for the 0.5 s base interval is shorter than for the 0.6 s base interval. No other differences reached the 5% level of statistical significance. Consistent with experiment 1, the central tendency effect on motor timing was not observed in the subsecond range.

The obtained ratios of SDs to their base intervals are shown in Figure 3E. A one-way repeated-measures ANOVA of these data revealed a significant main effect (*F*_(1.55,15.5)_ = 50.83, *p* < 0.0001, $\eta_{p}^{2}$ = 0.836). *Post hoc* paired *t*-tests with the Holm-Bonferroni correction revealed that the variability of reproduction for the 0.3 s base interval was larger than for all longer base intervals. That for the 0.4 s base interval was larger than for all longer base intervals, and that for the 0.5 s base interval was larger than the 0.6 s base interval (*p* < 0.05 for all comparisons). The reduction in the ratios of SDs to their base interval would be caused by interval-independent sources of variance originating from the motor system (Wing and Kristofferson, 1973; Ivry and Hazeltine, 1995).

## Experiment 3

In experiment 3, the base intervals were within the suprasecond range. The purpose of experiment 3 was similar to that of experiment 2.

### Materials and Methods

The experimental settings were the same as for experiments 1 and 2 except for the base intervals. The base intervals used in the experiment were 3.0, 3.1, 3.2 and 3.3 s. Ten individuals (8 female, average age 37.8 years, *SD* = 4.8 years) participated in the experiment. No data were excluded.

### Results and Discussion

#### Discrimination Performance

**Figure 4:**
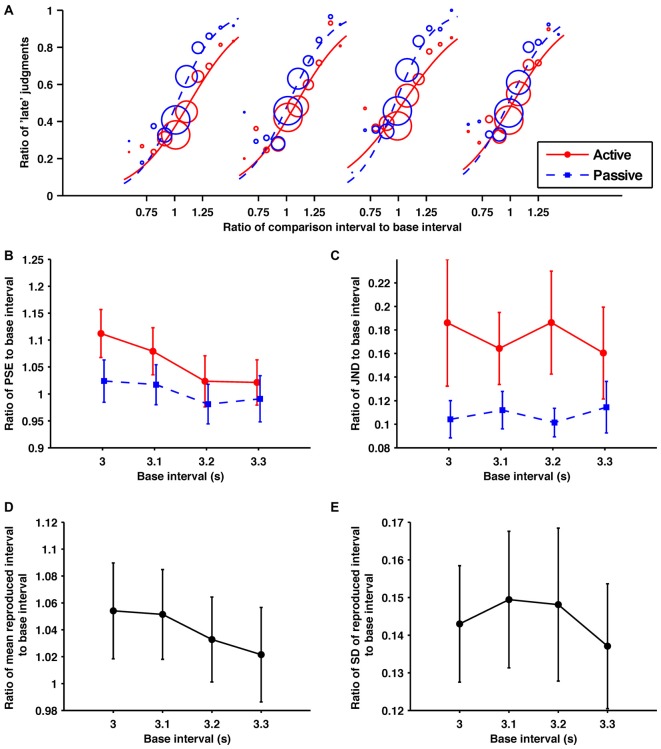
**Within the suprasecond range the self-produced time intervals were perceived as shorter and more variable. (A)** Probability of judgments calculated from the collected data from all participants, and the fitted psychometric curves in each condition/base interval. In order from the left, these fitted psychometric curves are for base intervals of 3.0, 3.1, 3.2 and 3.3 s. **(B)** Averaged data for the ratios of PSEs, **(C)** JNDs, **(D)** means of reproduction **(E)** SDs of reproduction to base interval as a function of base interval. The main effects of condition for PSEs and JNDs were significant. The main effects of base interval for PSEs and means of reproduction were significant. Error bars represent the standard error of the mean.

The fitted psychometric curves for the response data aggregated from all the participants are shown in Figure 4A. The obtained ratios of the PSEs to their base intervals are shown in Figure 4B. A 2 (condition; active and passive) × 4 (base interval; 3.0, 3.1, 3.2, 3.3 s) repeated-measures ANOVA of these data revealed a significant main effect of condition (*F*_1,9_ = 13.54, *p* = 0.005, $\eta_{p}^{2}$ = 0.601) and base interval (*F*_(1.30,11.7)_ = 9.46, *p* = 0.007, $\eta_{p}^{2}$ = 0.512). The interaction fell short of significance (*F*_(1.31,11.8)_ = 3.20, *p* = 0.092, $\eta_{p}^{2}$ = 0.262). Consistent with experiment 1, the comparison interval was shortened by temporal reproduction subjectively. *Post hoc* paired *t*-tests with the Holm-Bonferroni correction revealed that the ratio of PSE to its base intervals for the 3.0 s base interval was larger than for the 3.3 s base interval, and that for the 3.1 s base interval was larger than for the 3.2, and 3.3 s base intervals (*p* < 0.05 for all comparison). No other differences reached the 5% level of statistical significance. Consistent with experiments 1 and 2, the central tendency effect on perceptual timing was observed.

The obtained ratios of the JNDs to their base intervals are shown in Figure 4C. A 2 (condition) × 4 (base interval) repeated-measures ANOVA of these data revealed a significant main effect of condition (*F*_1,9_ = 5.28, *p* = 0.047, $\eta_{p}^{2}$ = 0.370). The main effect of the base interval and the interaction were not significant (*p* = 0.78, *p* = 0.29, respectively). Consistent with experiment 1, the temporal sensitivity was worsened by temporal reproduction.

#### Reproduction Performance

The obtained ratios of the means to their base intervals are shown in Figure 4D. A one-way repeated-measures ANOVA for these data revealed a significant main effect (*F*_(2.03,18.3)_ = 8.67, *p* = 0.002, $\eta_{p}^{2}$ = 0.490). A *post hoc* paired *t*-test revealed that the ratio of the mean to its base interval for the 3.0 s base interval was larger than for 3.3 s, and that for the 3.1 s base interval was larger than for the 3.3 s base interval (*p* < 0.05 for all comparison). No other differences reached the 5% level of statistical significance. Consistent with experiment 1, the central tendency effect on motor timing was observed in the suprasecond range.

The obtained ratios of the SDs to their base intervals are shown in Figure 4E. A one-way ANOVA was performed for these data. There was no significant main effect (*p* = 0.41).

## Experiment 4

To compare the performances in the sub- and suprasecond ranges directly, we conducted a similar experiment to previous experiments in the sub- and suprasecond ranges in a within-subject design. In addition to this, to assess the contextual effects, we performed experiment 4 in a constant context where a single base interval was presented in a session. In the constant context, the effect of temporal adaptation would be observed.

### Materials and Methods

The experimental settings were the same as for experiments 1, 2 and 3 except for the way the base intervals were presented. The base intervals used in the experiment were 0.5 and 3.2 s. The 0.5 and 3.2 s base interval conditions were employed on separate days. The order of the base interval conditions was counterbalanced. Seventeen individuals participated in this experiment. Three of the participants were excluded from the analysis, because their variability of temporal reproduction was extremely large even after the outliers were excluded (the ratio of the SD to its base interval was >0.5) in at least one of the 0.5 and 3.2 s base interval conditions. The data of the remaining 14 participants (11 female, average age 37.1 years, *SD* = 6.4 years) were analyzed.

### Results and Discussion

#### Discrimination Performance

**Figure 5:**
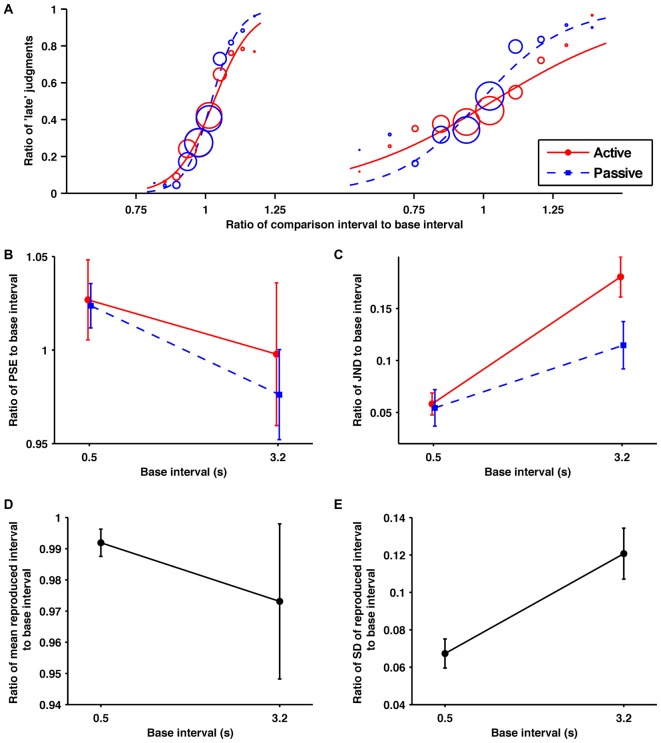
**The self-produced time intervals were perceived as more variable in the suprasecond range but not in the subsecond. (A)** Probability of judgments calculated from the data collected from all the participants, and the fitted psychometric curves in each condition/base interval. In order from the left, these fitted psychometric curves are for base intervals of 0.5 and 3.2 s. **(B)** Averaged data for the ratios of PSEs, **(C)** JNDs, **(D)** means of reproduction **(E)** SDs of reproduction to base interval as a function of base interval. The main effects of condition for JNDs were significant. The main effects of base interval for JNDs and SDs of reproduction were significant. The interaction for JNDs was significant. Error bars represent the standard error of the mean.

The fitted psychometric curves for the response data aggregated from all the participants are shown in Figure 5A. The obtained ratios of the PSEs to their base intervals are shown in Figure 5B. A 2 (condition; active and passive) × 2 (base interval; 0.5 and 3.2 s) repeated-measures ANOVA of these data was performed. There was no significant main effect of condition and base interval, and the interaction (*p* = 0.39, 0.34, 0.47). In contrast to experiments 1 and 3, the criterion was not biased consistently by temporal reproduction.

The obtained ratios of the JNDs to their base intervals are shown in Figure 5C. A 2 (condition) × 2 (base interval) repeated-measures ANOVA of these data revealed a significant main effect of condition (*F*_1,13_ = 6.40, *p* = 0.025, $\eta_{p}^{2}$ = 0.330), base interval (*F*_1,13_ = 30.28, *p* = 0.0001, $\eta_{p}^{2}$ = 0.700), and interaction (*F*_1,13_ = 4.79, *p* = 0.048, $\eta_{p}^{2}$ = 0.270). A *post hoc* paired *t*-test revealed that the temporal sensitivity was significantly worse in the active condition than in the passive condition for the 3.2 s base interval (*p* = 0.02) but not for the 0.5 s base interval (*p* = 0.78), which is consistent with results of experiments 2 and 3. Further, the *post hoc* analysis indicated that the temporal sensitivity was significantly worse for the 3.2 s base interval than for the 0.5 s base interval in both the active (*p* < 0.001) and passive conditions (*p* = 0.02), which is consistent with experiment 1.

#### Reproduction Performance

The obtained ratios of the means to their base intervals are shown in Figure 5D. A paired *t*-test revealed no significant difference between the 0.5 and 3.2 s base interval conditions (*t*_(13)_ = 0.71, *p* = 0.49, Cohen’s *d* = 0.28).

The obtained ratios of the SDs to their base intervals are shown in Figure 5E. A paired *t*-test revealed a significant difference between the 0.5 and 3.2 s base interval conditions (*t*_(13)_ = 4.14, *p* = 0.001, Cohen’s *d* = 1.29). This result indicates that the variability of motor timing is larger in the suprasecond range than in the subsecond range, which is consistent with experiment 1.

## General Discussion

This study has shown that temporal sensitivity is worse for self-produced time intervals than for passively presented time intervals, across the sub- and suprasecond ranges and within the suprasecond range, but not within the subsecond range. The impairment of temporal sensitivity was expected to be caused by the effect of timing processing for motor control. Previous studies have revealed an improvement in auditory time perception by body movements (Iordanescu et al., 2013; Manning and Schutz, 2013), which is the opposite direction to our results. Both studies have reported that body movements sharpen time perception for target intervals preceded action. Therefore, the effect of body movements that improve auditory temporal sensitivity would occur only when the body movements precede target intervals or would be covered by the deterioration effect of concurrent timing processing for motor control. Another major difference between these studies and our study is whether the target interval is determined by participant’s response. This might also be a reason for the inconsistent results.

The PSEs tended to be larger in the active condition than in the passive condition in experiments 1 and 3, which also cannot be explained by the effects of body movements. The criterion shift indicates the compression of subjective time or a response bias induced by a self-producing time interval. Compression of subjective time has often been associated with less attention being paid to time (for a review, see Block et al., 2010). Thus, the compression of subjective time by temporal reproduction can be interpreted as the result of less attention being paid to encoding the reproduced time interval. The compression of a self-produced time interval is clearly inconsistent with the finding of Hagura et al. (2012), which indicates that time intervals expand during the motor preparatory period. The inconsistency would be caused by a difference of motor tasks or the modality of the target interval. The task used by Hagura et al. (2012) required rapid and large movements of the arm and visual time perception, whereas the task used in our study required small accurately timed manual movements and auditory time perception. Further, the timing of movement is also a major difference between this study and our study. In their study, which used a reaction time task, the timing of movement was just after the end of the target interval, whereas in our study using a temporal reproduction task, the timing of movement was just before the end of the target interval. In addition, the relationship between the timing and movement would make the participants feel that the target intervals were produced by their own action in our study but not in Hagura’s study. This might also be a reason for the compression or response bias.

The pattern of impairment in experiment 2, 3 and 4 would reflect the fact that timing processing for the subsecond range is automatic (i.e., fewer attentional demands), whereas that for the suprasecond range is cognitively mediated (i.e., more attentional demands; Lewis and Miall, 2003b). Consistent with our results, previous research has reported that a concurrent non-temporal cognitive task interferes with temporal discrimination and motor timing for the suprasecond range but not the subsecond range, which is evidence for the automaticity of subsecond-timing (Rammsayer and Lima, 1991; Miyake et al., 2004; Rammsayer and Ulrich, 2011; Maes et al., 2015; but see Rammsayer and Ulrich, 2005). The lack of the interference effect within the subsecond range would be caused by the automaticity of either temporal reproduction or the encoding time interval (i.e., motor or perceptual timing), or both.

On the other hand, the impairment of temporal sensitivity by a temporal reproduction task was observed not only in the suprasecond range but also in the subsecond range in experiment 1. Although we did not expected the impairment in the subsecond range, this could be also explained by the framework of the distinct timing mechanisms used for the sub- and suprasecond ranges. When the target intervals are across the sub- and suprasecond ranges, participants must switch over these timing mechanisms. The switching might eliminate the automaticity of temporal discrimination or reproduction.

The subjective compression by temporal reproduction was not observed in the suprasecond range in experiment 4 where the same target intervals were always presented in the experimental session. Whether the subjective compression by self-producing occurs would be relevant to temporal context effects and/or temporal adaptation, although we have no straightforward explanation for the contradiction. As noted above, temporal context affects subjective time, such as the central tendency effect. The compression might be due to the interaction between temporal context and temporal reproduction rather than temporal reproduction alone. Another explanation is that adaptation caused by repeatedly presenting the same interval would lead to a lack of compression. A reduction in neural activity caused by adaptation has been found in the human parietal cortex (Hayashi et al., 2015). Psychophysically, it was found to be a phenomenon associated with adaptation in the sub- and suprasecond ranges (Becker and Rasmussen, 2007; Heron et al., 2012; Shima et al., 2016). The phenomenon, which consists of the repetitive exposure of a longer interval, makes a subsequent short interval even shorter and the repetitive exposure of a shorter interval makes a subsequent long interval even longer. This must increase the subjective deviation of the comparison interval from the base interval. The enlargement of the subjective deviation might weaken the compression effect.

We have observed that the variability of motor and perceptual timing increased from the subsecond range to the suprasecond range in experiments 1 and 4. This suggests distinct timing mechanisms for the sub- and suprasecond ranges. The scalar timing model, which assumes a unitary timing mechanism, predicts a constant ratio of variability to timed interval, which is referred to as the scalar property (Gibbon et al., 1984; Gibbon, 1991). An increase in variability indicates a violation of the scalar property. Therefore, our results support the idea of distinct timing mechanisms for the sub- and suprasecond ranges. Consistent with our results, the violation of the scalar property around 1–2 s has been reported (Gibbon et al., 1997; Grondin, 2012).

Additionally, our results suggest that the degree of overlap between sub- and suprasecond timing mechanisms is smaller for motor than for perception. The increased motor variability was rapid whereas that of perceptual timing was gradual from the subsecond range to the suprasecond range. The notion of the overlapped mechanisms for the sub- and suprasecond ranges is supported by a meta-analysis of neuroimaging studies (Wiener et al., 2010, 2011) and a confirmatory factor analysis (Rammsayer and Troche, 2014). Bangert et al. (2011) have already shown that the violation of the scalar property is clearer for motor timing than for perceptual timing within 0.3–1.87 s. We have shown that the difference of the degree of violation between motor and perceptual timing is valid in the 0.5–4.2 s range.

Subsecond timing mechanisms for motor and perception would be distinct, whereas suprasecond ones would be common. We have generally observed the central tendency effect, which consists of the overestimation of shorter intervals and the underestimation of longer intervals. Nevertheless, the central tendency effect did not occur with motor timing in the subsecond range. Repp (2002) has reported that the contextual effect is weaker for motor timing than for perceptual timing in the subsecond range and argued that the difference between the contextual effects for perceptual and motor timing reflect the fact that perceptual timing requires an additional process for conscious awareness. Our results indicate that this notion by Repp (2002) holds for the subsecond range, but not for the suprasecond range.

## Author Contributions

KM, and MK designed the experiment and wrote the article. KM performed the experiment, and analyzed the data.

## Conflict of Interest Statement

The authors declare that the research was conducted in the absence of any commercial or financial relationships that could be construed as a potential conflict of interest.
